# Impaired Glucose Homeostasis in a Tau Knock-In Mouse Model

**DOI:** 10.3389/fnmol.2022.841892

**Published:** 2022-02-16

**Authors:** Hamza Benderradji, Sarra Kraiem, Emilie Courty, Sabiha Eddarkaoui, Cyril Bourouh, Emilie Faivre, Laure Rolland, Emilie Caron, Mélanie Besegher, Frederik Oger, Theo Boschetti, Kévin Carvalho, Bryan Thiroux, Thibaut Gauvrit, Emilie Nicolas, Victoria Gomez-Murcia, Anna Bogdanova, Antonino Bongiovanni, Anne Muhr-Tailleux, Steve Lancel, Kadiombo Bantubungi, Nicolas Sergeant, Jean-Sebastien Annicotte, Luc Buée, Didier Vieau, David Blum, Valérie Buée-Scherrer

**Affiliations:** ^1^Univ. Lille, Inserm, CHU Lille, U1172 LilNCog—Lille Neuroscience & Cognition, Lille, France; ^2^Alzheimer & Tauopathies, LabEx DISTALZ, Lille, France; ^3^Univ. Lille, INSERM, CNRS, CHU Lille, Institut Pasteur de Lille, Inserm U1283-UMR8199—EGID, Lille, France; ^4^Development and Plasticity of the Neuroendocrine Brain, Lille, France; ^5^Univ. Lille, CNRS, Inserm, CHU Lille, Institut Pasteur de Lille, US 41—UMS 2014—PLBS, Animal Facility, Lille, France; ^6^Univ. Lille, Inserm, CHU Lille, Institut Pasteur de Lille, U1011-EGID, Lille, France; ^7^Univ. Lille, CNRS, Inserm, CHU Lille, Institut Pasteur de Lille, US 41—UMS 2014—PLBS, BioImaging Center Lille, Lille, France; ^8^Univ. Lille, Inserm, CHU Lille, Institut Pasteur de Lille, U1167—RID-AGE—Facteurs de risque et déterminants moléculaires des maladies liées au vieillissement, Lille, France

**Keywords:** tau, glucose homeostasis, energy metabolism, mouse model, high-fat

## Abstract

Alzheimer’s disease (AD) is the leading cause of dementia. While impaired glucose homeostasis has been shown to increase AD risk and pathological loss of tau function, the latter has been suggested to contribute to the emergence of the glucose homeostasis alterations observed in AD patients. However, the links between tau impairments and glucose homeostasis, remain unclear. In this context, the present study aimed at investigating the metabolic phenotype of a new tau knock-in (KI) mouse model, expressing, at a physiological level, a human tau protein bearing the P301L mutation under the control of the endogenous mouse *Mapt* promoter. Metabolic investigations revealed that, while under chow diet tau KI mice do not exhibit significant metabolic impairments, male but not female tau KI animals under High-Fat Diet (HFD) exhibited higher insulinemia as well as glucose intolerance as compared to control littermates. Using immunofluorescence, tau protein was found colocalized with insulin in the β cells of pancreatic islets in both mouse (WT, KI) and human pancreas. Isolated islets from tau KI and tau knock-out mice exhibited impaired glucose-stimulated insulin secretion (GSIS), an effect recapitulated in the mouse pancreatic β-cell line (MIN6) following tau knock-down. Altogether, our data indicate that loss of tau function in tau KI mice and, particularly, dysfunction of pancreatic β cells might promote glucose homeostasis impairments and contribute to metabolic changes observed in AD.

## Introduction

Neurofibrillary degeneration, made of aggregates of hyper- and abnormally phosphorylated tau proteins (tau pathology) is a neuropathological hallmark of tauopathies including Alzheimer’s disease (AD; Sergeant et al., 2008; Colin et al., 2020). In the latter, the spatio-temporal progression of tau pathology has been tightly correlated to cognitive deficits, supporting an instrumental role (Colin et al., 2020). Whether this relates to a toxic gain or a pathological loss of tau function remains debated (Maeda and Mucke, 2016). Indeed, on the one hand, transgenic models developing tau pathology exhibit synaptic impairments and cognitive deficits (i.e., Van der Jeugd et al., 2013). On the other hand, tau knock-out or knock-down models display similar alterations (Ahmed et al., 2015; Biundo et al., 2018; Velazquez et al., 2018). These latter observations particularly support that tau, essentially expressed by neurons in the nervous system, exerts physiological functions whose loss promotes neuron-autonomous dysfunctions. This might relate to the ability of tau to control microtubule dynamics but possibly to other mechanisms, providing that tau is now acknowledged to be more than a microtubule-associated protein (Sotiropoulos et al., 2017). From a general perspective, the physiological functions of tau remain ill-defined.

Diabetes and impaired glucose tolerance are important risk factors for AD (Reitz et al., 2011; Livingston et al., 2017). Hyperglycemia, even without the development of diabetes, represents a risk factor for memory decline and AD (Crane et al., 2013). Diabetes was also reported to be an independent risk factor in patients with frontotemporal lobar degeneration (FTLD; Golimstok et al., 2014). In agreement, inducing glucose homeostasis impairments and diabetes exacerbate learning and memory defects as well as underlying pathology in different models reproducing the amyloid and tau lesions of AD (Takeda et al., 2010; Leboucher et al., 2013; for review see Wijesekara et al., 2018a). Puzzlingly, while impaired glucose homeostasis has been suggested to increase AD risk and associated lesions and particularly tau pathology, AD patients have been reported to exhibit altered glucose metabolism (Bucht et al., 1983; Fujisawa et al., 1991; Craft et al., 1992; Matsuzaki et al., 2010; Calsolaro and Edison, 2016; Tortelli et al., 2017) and to display an increased prevalence to develop type 2 diabetes (Janson et al., 2004; for review see Gratuze et al., 2018). It has been also reported that patients presenting the most common clinical phenotype of FTLD i.e., the behavioral variant or bvFTLD, among which one-half anatomo-pathologically present with tau aggregates (Pressman and Miller, 2014), exhibit increased fasting insulin levels and HOMA-IR index, a marker of insulin resistance, suggesting impaired glucose metabolism (Ahmed et al., 2014). The origin of these metabolic changes remains however unclear. However, at least for AD, the presence of tau pathology was described in brain regions known to control peripheral metabolism such as the hippocampus and hypothalamus (Schultz et al., 1999; Ishii and Iadecola, 2015; Soto et al., 2019) but also, surprisingly, in insulin-producing pancreatic β cells (Martinez-Valbuena et al., 2019).

We recently provided evidence, using a model of constitutive deletion, that tau is important for the control of peripheral energy homeostasis (Marciniak et al., 2017). We particularly showed that tau knock-out mice exhibit glucose homeostasis impairments, characterized by hyperinsulinemia and impaired glucose tolerance, that have been later replicated by other colleagues (Wijesekara et al., 2018b, 2021). In agreement, Wijesekara et al. (2021) recently demonstrated that human tau expression reversed glucose intolerance observed in tau knock-out mice. Further, we associated the H1 tau haplotype with glucose homeostasis in humans (Marciniak et al., 2017). These observations raised the hypothesis that, overall, the pathological loss of tau function promotes glucose homeostasis impairments seen in AD patients. To address this question, in the present study, we have investigated the peripheral metabolic outcomes in a new knock-in model of tau loss-of-function, expressing mutated (P301L) human tau protein under the control of the endogenous murine *Mapt* promoter. Overall, our data report the vulnerability of tau knock-in mice to glucose metabolism alterations, supporting the prime function of tau dysfunctions to glucose dyshomeostasis described in AD.

## Materials and Methods

### Human Samples

Human tissues were obtained in accordance with French bylaws (Good Practice Concerning the Conservation, Transformation, and Transportation of Human Tissue to be Used Therapeutically, published on 29 December 1998). Permission to use human tissues was obtained from the French Agency for Biomedical Research (Agence de la Biomedecine, Saint-Denis la Plaine, France, protocol no. PFS16-002) and the Lille Neurobank (DC-2008-642). To monitor tau isoforms in human islets, we used 3 mRNA samples obtained from TEBU-Bio (France). As control of tau isoform expression in the brain, we used mRNA extracted from the cortical area of one 29-year-old male individual who had donated his body to science. To evaluate tau expression in human islets by immunohistochemistry, we used pancreatic sections from a 77-year-old male obtained from Biochain^1^ (T2234188, Hayward, CA).

### Experimental Animals and Diet

**Figure 1:**
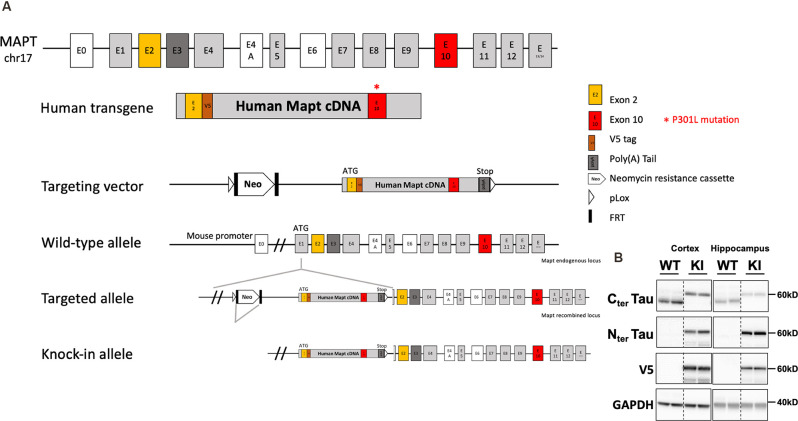
Generation and tau expression in the tau knock-in mouse model. **(A)** Generation strategy of tau knock-in (KI) mice. MAPT human transgene is composed of 10 exonic regions with P301L mutation on exon 10. A V5 epitope tag is inserted after exon 2. STOP codon is inserted followed by exogenous poly(A) Tail. The transgene is under the control of the Mapt mouse endogenous promoter. Insertion of the targeting vector was mediated by cre-loxP recombination (pLox sites shown as empty arrows). A floxed neomycin resistance (Neor) cassette used for positive selection was removed from the targeted allele by FRT (FRT sites shown as black rectangles) recombination sites. Positions and sizes of exons and introns are not to scale. **(B)** Representative expression of tau and V5 in the cortex and hippocampus of tau KI mice and WT littermates (two showed mice out of eight/genotype).

Tau knock-in mice (tau KI; C57BL6/J background) were generated by knock-in targeted inserting way, into the murine locus *Mapt* gene, of a cDNA encoding human 1N4R isoform mutated at P301L and tagged with a V5 epitope (GKPIPNPLLGLDST; this epitope tracks transgene expression) in exon 1 after initiation codon of protein translation (ATG). A Stop codon is present at the end of the human transgene as well as a poly(A) tail (Genoway, France; Figure 1A). Human tau expression in this KI model was assessed by Western blot. To obtain animals of interest, we crossed heterozygous tau KI male mice with heterozygous females tau KI animals to generate the homozygous tau KI mice and their littermate WT controls used for experiments. It is noteworthy that the body weight at weaning was similar in tau KI mice as compared to WT littermate (not shown). Animals were maintained in standard animal cages under conventional laboratory conditions (12-h/12-h light/dark cycle, 22°C), with ad libitum access to food and water. The animals were maintained in compliance with European standards for the care and use of laboratory animals and experimental protocols approved by the local Animal Ethical Committee (agreement APAFIS# 12787-2015101320441671 v9 from CEEA75, Lille, France). Tau KI mice and WT littermates were fed with CHOW diet (SAFE D04; for composition see: https://safe-lab.com/safe-wAssets/docs/product-data-sheets/diets/safe_d04ds.pdf) or High-Fat Diet (HFD; 58% kCal from fat; Research Diets D12331; for composition see: https://researchdiets.com/formulas/d12331) from 2 months of age. Body weights were measured weekly. At the completion of the experiment (i.e., following 12w of diet), mice were about 5-month-old. The experimental workflow for metabolic observations is provided on Supplementary Figure 1 and experiments are detailed below. Comparison of the metabolic phenotype of the KI mice with littermate WT was performed under chow diet in a first experiment; the effect of the HFD diet was evaluated in a second experiment. The HFD used is similar to what we published previously (Leboucher et al., 2019). This HFD was subjected to an initial evaluation of metabolic properties in WT littermate mice of the KI strain to ensure the ability to promote glucose intolerance.

### Metabolic Cages

Spontaneous feeding, locomotor activity (total beam breaks/hour), respiratory exchange ratio, and O_2_ consumption were monitored continuously for 24 h using metabolic cages (Phenomaster, TSE Systems, Germany). Food intake was measured by the integration of weighing sensors fixed at the top of the cage from which the food containers were suspended into the home cage. Locomotor activity was assessed using a metal frame placed around the cage. Evenly spaced infrared light beams are emitted along the x axis. Beam interruptions caused by movements of the animals are sensed and registered at high resolution. The sensors for detection of movement operate efficiently under both light and dark phases, allowing continuous recording. Metabolic rates were (Respiratory exchange ratio, O_2_ consumption) measured by indirect calorimetry. Mice were housed individually and acclimated to the home cage for 72 h prior to experimental measurements.

### Biochemical Plasma Parameters

Blood was collected at the tail vein after 6 h of morning fasting. Within 30 min of the collection of blood samples, blood was centrifuged at 1,500 *g* for 15 min at 4°C. Plasma was separated, transferred to 1.5 ml Eppendorf tubes, and stored at −80°C until analysis. Plasma concentrations of insulin were measured using the mouse insulin ELISA kit (Mercodia AB, 10-1247-01; no cross-reactivity with proinsulin) following the manufacturer’s instructions. Plasma concentrations of adiponectin were measured using a mouse Adiponectin ELISA kit (Invitrogen, KMP0041) following the manufacturer’s instructions.

### Metabolic Tolerance Tests

Intraperitoneal glucose tolerance tests (IPGTT) were assessed following 6 h of morning fasting. D (+) glucose (1 g/kg; Sigma-Aldrich) was injected intraperitoneally. Blood glucose was then measured at 0, 15, 30, 60, 90, and 120 min following injection. For the pyruvate tolerance test (PTT), mice were fasted overnight and given an intraperitoneal injection of sodium pyruvate (2.0 g/kg) dissolved in sterile saline. Blood glucose was then measured at 0, 15, 30, 60, 90, and 120 min following injection. For fasting-refeeding experiments, mice were fasted overnight (16 h of fast) and re-fed. Blood glucose was measured at 16 h (overnight) of fasting and during the 1st, 2nd, and 4th h after re-feeding. Blood glucose was measured at the tail vein after 6 h of morning fasting, an overnight fasting, and in fed or refeeding conditions using One Touch Verio Flex glucometer (LifeScan).

### Tissue Fixation, Immunohistochemistry and Imaging

Animals were sacrificed by cervical dislocation. Following dissection, pancreases were laid flat in cassettes, fixed for 4 h in 4% paraformaldehyde, dehydrated, and embedded in paraffin. Longitudinal serial sections (5 μm) were processed for immunofluorescent (IF) analysis. Tissue sections from human adult normal pancreas were obtained from Biochain (T2234188, Hayward, CA). Immunohistochemistry of the human pancreas was performed on 5 μm sections embedded in paraffin. The sections were de-paraffinized in three changes of toluene (5 min each) and re-hydrated in decreasing serial solutions of ethanol (100%, 95%, and 70%) and PBS. Sections were submitted to heat-induced antigen retrieval in citrate-buffer (10 mM citrate acid, 0.05% Tween 20 in distilled water), using microwave: two cycles for 5 and 10 min at power level 520 W and 160 W, respectively, followed by a 2 min break, cooled to room temperature for 20 min. Pancreatic tissue samples were incubated with blocking solution (5% goat serum and 1% BSA in PBS) for 1 h at RT, and washed once with PBS, then incubated with the primary anti-tau antibodies (laboratory-made mouse monoclonal IgG1 tau C-ter 9F6 raised against amino-acids (aas) 427–441, homemade mouse monoclonal IgG2b tau 9H12 raised against aas 162–175, for human sections a homemade mouse polyclonal IgG tau C-ter 993S5 raised against aas 394–408; see Supplementary Figure 2) diluted at 1:200 in antibody buffer (PBS, 1% BSA) overnight at +4°C. After washing in PBS, slides were incubated with the detecting secondary antibodies conjugated to Alexa Fluor 568 (IgG H + L, Highly Cross-Adsorbed Goat anti-Mouse, Invitrogen, A-1103, Darmstadt, Germany) diluted at 1:200 in antibody buffer for 1 h at RT. For detecting human tau in tau KI mice sections, a homemade rabbit monoclonal tau N-ter (hTauE1, raised against aas 12–21; Supplementary Figure 2) was used, diluted at 1:200 in antibody buffer (PBS, 1% BSA) 48 h at + 4°C. Amplified immunohistochemistry processes were used. After washing in PBS, slides were incubated with Goat Anti-Rabbit IgG biotinylated secondary antibody (BA-1000-1.5), washed, and incubated with fluorophore-coupled streptavidin (Alexa Fluor^TM^ 647 Conjugate, S32357) diluted at 1:600 in PBS. For detection of glucagon and insulin, slides were incubated with either a recombinant monoclonal Rabbit anti-glucagon antibody (Abcam, ab92517) diluted at 1:500 or a Polyclonal Guinea Pig Anti-insulin antibody ready-to-use (Agilent, IR00261-2) overnight at +4°C, followed by the secondary antibodies conjugated to Alexa Fluor 488 [for glucagon: IgG H + L, Highly Cross-Adsorbed Goat anti-Rabbit, Invitrogen, A32731, Darmstadt, Germany; for insulin: IgG H + L, Highly Cross-Adsorbed Goat anti-Guinea Pig, A-11073, Darmstadt, Germany] diluted at1:200 in antibody buffer (PBS, 1% BSA) for 1 h at RT. Nuclear counterstaining was performed using DAPI (Invitrogen). Sections were quenched for autofluorescence using the Vector TrueVIEW Autofluorescence Quenching Kit (Vector Laboratories, Burlingame, CA, USA). Slides were mounted using Dako Fluorescence Mounting Medium (Agilent Technologies, California, USA). Immunofluorescence-stained slides were imaged using a Zeiss Spinning disk confocal microscopy with a 40× oil-immersion lens (NA 1.3 with an optical resolution of 176 nm). Images were processed with ZEN software (Carl Zeiss, version 14.0.0.201, Germany). Colocalizations between islet signals given using tau antibodies vs. insulin or glucagon were determined through Pearson’s overlap coefficient using Image J (Adler and Parmryd, 2010).

### Identification of 3R and 4R Tau Isoforms

Following mRNA extraction, one microliter of the RT-product was used as the template for subsequent PCR amplification. All PCR primers used in this study are reported in (Supplementary Table 1). Regarding mouse tau exon 10 splicing, we performed a nested PCR with TMF1/TMR1 primers to ensure the specificity of the PCR products obtained and a second PCR using TMF1/TRR2 primers. As internal controls, we used mouse or human cortex samples. The TMF1/TRR2 PCR products obtained were resolved in an 1.75% agarose gel in TAE buffer (40 mM Tris, 20 mM acetic acid, 2 mM EDTA, pH 8.5).

### Morphometric Analysis of Pancreatic Islets

Longitudinal pancreatic sections were cut at a 5 μm thickness, collected at 250 μm intervals, and plated on glass slides. This resulted in the collection of sections of 10 depths per pancreas. The sections were then proceeded as previously described (Rabhi et al., 2016). Sections were incubated with anti-glucagon and anti-insulin antibodies, followed by the secondary antibodies conjugated to Alexa Fluor 568 [IgG (H + L) Highly Cross-Adsorbed Goat anti-Rabbit, A-11008, Darmstadt, Germany], and Alexa Fluor 488 [IgG (H + L) Highly Cross-Adsorbed Goat anti-Guinea Pig, A-11073, Darmstadt, Germany], respectively. All images were acquired on a ZEISS Axio Scan.Z1 slide scanner (Carl Zeiss Microscopy GmbH, Germany) at ×20 magnification (resolution of 0.5 μm/pixel) and uploaded into a Spectrum digital slide interface. Images of whole pancreatic sections acquired were analyzed by a macro-based automated approach. First, pancreatic islets were detected by an automated approach using ImageJ software (Scion Software) based on immunofluorescence signal of insulin and glucagon. Then, to appreciate the relative mass of β and α cells in each detected pancreatic islet, the surface area of both insulin and glucagon positive cells was determined using the following equations:

B-cell surface area:


$$\frac{\left(\sum_{i\;=\;0}^{n}i=\%\text{ of insulin signal}\right)}{\left(\sum_{i\;=\;0}^{n}i\;\%\text{ of insulin and glucagon signals}\right)\;\times \;100}$$

α cells surface area:


$$\frac{\left(\sum_{i\;=\;0}^{n}i=\%\text{ of glucagon signal}\right)}{\left(\sum_{i\;=\;0}^{n}i\;\%\text{ of insulin and glucagon signals}\right)\;\times \;100}$$

### Cell Culture, siRNA Knock-Down, and Glucose-Stimulated Insulin Secretion (GSIS)

The mouse pancreatic β-cell line Min6 (AddexBio) was cultured in DMEM (Gibco) with 15% fetal bovine serum, 100 mg/ml penicillin-streptomycin, and 55 μM β-mercaptoethanol (Sigma, M6250). Cells were transfected with non-targeting siRNA mouse negative controls (siCont, D-001810-0X) and siTau (L-061561-01-0005, SMARTpool, Dharmacon) using Dharmafect1 (T-2001-03, GE Dharmacon) and GSIS experiments were performed 48 h later. For GSIS, following a 1 h preincubation in Krebs-HEPES-bicarbonate buffer (KHB; 140 mM NaCl, 3.6 mM KCl, 0.5 mM NaH_2_PO_4_, 0.2 mM MgSO_4_, 1.5 mM CaCl_2_, 10 mM HEPES, 25 mM NaHCO_3_) with 2.8 mM glucose, GSIS was assessed by static incubation of siCont and siTau transfected Min6 cells in KHB with 2.8 mM or 20 mM glucose for 1 h at 37°C. Mature insulin secreted into the media and total mature insulin content were quantified through insulin ELISA (Mercodia AB; no cross-reactivity with proinsulin) following the the manufacturer’s instructions.

#### Pancreatic Islet Isolation and GSIS

Mouse islets were isolated by type V collagenase digestion (Sigma-Aldrich C9263, 1 mg/ml h) of the pancreas for 10 min at 37°C. After separation in a density-gradient medium (Histopaque-1119; Sigma-Aldrich), islets were handpicked. They were then cultured for 18–20 h at 37°C in a 95% air/5% CO_2_ atmosphere in RPMI 1640 (Thermo Fisher Scientific) containing 10% FBS and 100 μg/ml penicillin-streptomycin. GSIS experiments were performed as previously described (Rabhi et al., 2016). Briefly, approximately 30 islets were exposed to 2.8 mM glucose and 16.7 mM glucose in Krebs-Ringer buffer supplemented with HEPES (Sigma, 83264) and 0.5% fatty-acid free BSA (Sigma, A7030). Insulin released in the medium and total insulin content were measured using the mouse insulin ELISA kit (Mercodia AB; no cross-reactivity with proinsulin) following manufacturer’s instructions.

#### Western Blot Analysis

Cortical brain and liver tissues, sampled at mouse sacrifice, were homogenized in a buffer Tris Base 10 mM; Sucrose 10%; pH = 7.4 with protease inhibitors (1 tablet for 10 ml solution—Sigma^®^ Complete Mini EDTA Free). Protein amounts were evaluated using the BCA assay (Pierce^TM^ BCA ProteinAssay Kit). Protein lysates were then diluted with LDS (Lithium Dodecyl Sulfate) 2× supplemented with reducing agents (NuPAGE^®^), and then separated on 18-well 4–12% acrylamide gel (Criterion XT, Biorad). Twenty microgram of total proteins for cortex as the hippocampus and 40 μg for liver were loaded per well. Proteins were transferred onto nitrocellulose membranes, which were saturated with 5% nonfat dry milk or Bovine Serum Albumin in Tris 15 mmol/L, pH 8; NaCl 140 mmol/L and 0.05% Tween then incubated with primary (listed below) and secondary antibodies (PI-1000-1 Goat Anti-Rabbit IgG Antibody (H + L), Peroxidase, Vector laboratories). Signals were visualized using chemiluminescence HRP substrate ECL kit (Amersham ECL Detection Reagents) and Amersham ImageQuant 800 imaging system (Cytiva). Results were normalized to GAPDH used as loading control, and quantifications were performed using ImageJ software. Anti-tau antibodies used for Western blot were Cter 9F6 and human tau-specific antibody Nter hTauE1 (Supplementary Figure 2). An antibody raised against V5 tag (GKPIPNPLLGLDST) that was inserted on human transgene (catalog no. AB3792 Anti V5 Epitope Tag (Rabbit polyclonal; Millipore) has been used to specifically label the human transgene. Phospho-Akt(S473) and Akt(pan) antibodies (Cell signaling) were used on liver tissue. Loading control anti-GAPDH antibody (catalog no. G9545-200UL, Sigma^®^).

#### mRNA Extraction and Quantitative Real-Time RT-PCR

Total RNAs from human (*N* = 3) and mouse (WT, *N* = 3) isolated pancreatic islets, as well mouse cortex were extracted from tissues using the RNeasy Lipid Tissue Kit (Qiagen, Courtaboeuf, France) following the manufacturer’s instructions. Samples were quantified with a NanoDrop ND-1000. Five-hundred nanograms of total RNA were reverse-transcribed using the High-Capacity cDNA reverse transcription kit (Applied Biosystem, Saint-Aubin, France). Quantitative real-time RT-PCR analysis was performed on an Applied Biosystems^TM^ StepOnePlus^TM^ Real-Time PCR Systems using TaqMan^TM^ Gene Expression Master Mix (Life Technologies Corp., Grand Island, NY). The thermal cycler conditions were as follows: 95°C for 10 min, then 40 cycles at 95°C for 15 s and 60°C for 1 min. Predesigned Taqman^TM^ gene expression assays (Life Technologies Corp., Grand Island, NY) were used for mouse *Mapt* (Mm00521988_m1). Peptidylprolyl isomerase A (PPIA, Mm02342430_g1) expression was assessed as a reference housekeeping gene for normalization. Amplifications were carried out in duplicates and the relative expression of target genes was determined by the ΔΔCt method.

#### Statistics

Results are expressed as mean ± SEM. Statistics were performed using either Student’s t-test as well as One or Two-way analysis of variance (ANOVA), followed by a *post hoc* Tukey’s test. We used Kruskall-Wallis when data failed a Kolmogorov-Smirnov or a Shapiro-Wilk normality test. Statistics were performed using Graphpad Prism Software. P values <0.05 were considered significant.

## Results

In the present study, we have performed a metabolic evaluation, focusing on glucose homeostasis, of tau KI mice in which the human 1N4R isoform mutated at P301L has been inserted at the locus of the mouse *Mapt* gene. Human tau expression was validated by western-blot analysis (Figure 1B). Noteworthy, similarly to other KI strains reported (Hashimoto et al., 2019; Saito et al., 2019), the present model does not exhibit tau aggregation at the age studied (2–5 months of age; not shown). This allowed us to evaluate the impact of an expression of soluble mutated (dysfunctional) tau proteins in absence of overexpression.

### Metabolism of Tau KI Mice Is Not Impaired Under Chow Diet

In a first attempt, we investigated the phenotype of male animals under a chow diet. Among all parameters measured i.e., food intake, ambulatory activity, respiratory exchange ratio (RER), and energy expenditure—indirectly represented by oxygen consumption VO_2_- using metabolic cages (Supplementary Figures 3A–D), as well as fed and fasted glycemia, plasma insulin, body weight gain over a 3-month period (from 2 to 5 months of age), glucose tolerance or rectal temperature, no significant change could be observed (Supplementary Figures 3E–J). Therefore, under chow diet, tau KI mice did not exhibit altered basal energy homeostasis or glucose metabolism.

### Impaired Glucose Metabolism in Tau KI Mice Under High-Fat Diet

In order to uncover a possible metabolic disorder related to the expression of the mutated human tau protein, we challenged tau KI male mice and their littermate controls with HFD for a period of 12 weeks, to promote the development of metabolic changes, approaching features of human metabolic syndrome or type 2 diabetes (Winzell and Ahren, 2004). HFD was given from 2 months of age, a time-point at which animals do not display any metabolic change in chow diet condition (Supplementary Figure 3). The time-line for metabolic investigations is given in Supplementary Figure 1.

**Figure 2:**
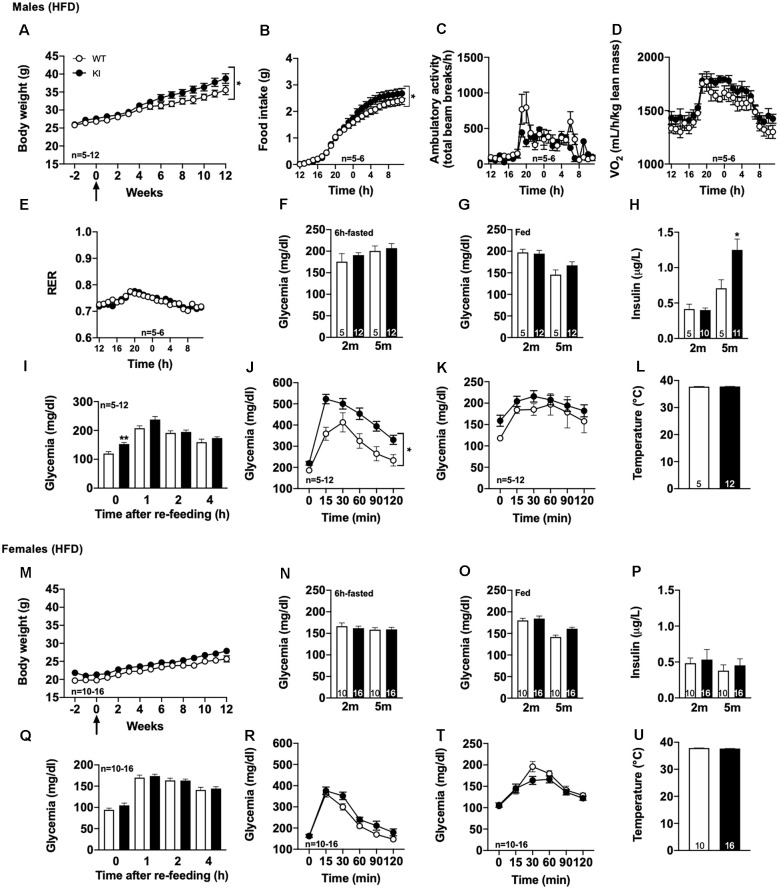
Metabolic phenotyping of tau KI mice under High Fat diet (HFD; given from 2 to 5 months of age). **(A–L)**: Males. **(A)** Body weight gain of WT and tau KI mice under HFD from 2 to 5 months of age (Two-Way ANOVA; *F*_(14,210)_ = 1.807, *p < 0.05 vs. WT). **(B–F)** Metabolic cage evaluation of tau KI male mice: **(B)** 24 h-cumulative food intake (g) (Two-Way ANOVA; *F*_(23,207)_ = 2.401, *p < 0.05 vs. WT). **(C)** 24 h spontaneous locomotor activity (total beam breaks/h). **(D)** 24 h-respiratory exchange ratio (RER = VCO_2_/VO_2_). **(E)** 24 h-O_2_ consumption. **(F)** Glycemia after 6 h of fasting before (2 m) and at the completion of HFD (5 m; NS, One-Way ANOVA). **(G)** Glycemia in fed condition (9 a.m) before (2 m) and at the completion of HFD (5 m; NS, One-Way ANOVA). **(H)** Insulinemia after 6 h of fasting before (2 m) and at the completion of HFD (5 m; One-Way ANOVA followed by Tukey’s post-hoc test; *F*_(3,27)_ = 13.34 *p* < 0.0001; *p < 0.05 vs. WT). **(I)** Glycemic variations during the 1st, 2nd and 4th h following re-feeding after 16 h of fasting at the completion of the HFD, i.e., 5 months of age (One-Way ANOVA followed by Tukey’s post-hoc test; *F*_(7,60)_ = 19.83 *p* < 0.0001; *p < 0.05 vs. WT *p < 0.05 vs. WT for glycemia after 16 h). **(J)** Intraperitoneal glucose tolerance test (IPGTT) at completion of the HFD, i.e., 5 months of age (*p < 0.05, Two-Way ANOVA). **(K)** Pyruvate tolerance test (PTT) at completion of the HFD, i.e., 5 months of age (NS, Two-Way ANOVA). **(L)** Rectal temperature at the completion of the HFD, i.e., 5 months of age (NS, Student’s t-test). **(M–U)**: Females. **(M)** Body weight gain of WT and tau KI mice under HFD from 2 to 5 months of age (NS). **(N)** Glycemia after 6 h of fasting before (2 m) and at the completion of HFD (5 m; NS). **(O)** Glycemia in fed condition 9 a.m. before (2 m) and at the completion of HFD (5 m; NS). **(P)** Insulinemia after 6 h of fasting before (2 m) and at the completion of HFD (5 m; NS). **(Q)** Glycemic variations during the 1st, 2nd and 4th h following re-feeding after 16 h of fasting at the completion of the HFD, i.e., 5 months of age (NS). **(R)** Intraperitoneal glucose tolerance test (IPGTT) at the completion of the HFD, i.e., 5 months of age (NS). **(T)** Pyruvate tolerance test (PTT) at the completion of the HFD, i.e., 5 months of age (NS). **(U)** Rectal temperature at the completion of the HFD, i.e., 5 months of age (NS). Results are expressed as mean ± SEM. WT mice are indicated as white circles/bars, tau KI mice as black circles/bars.

First, we observed that the body weight gain was significantly, even moderately, increased in tau KI as compared to littermate WT (Figure 2A, reaching, at the completion of the experiment, i.e., after 12 weeks of HFD at 5 months of age, 39.0 ± 3.1% above the initial body weight (2 months of age) vs. 28.9 ± 3.3% in WT mice (*p* < 0.05, Student’s t-test). In accordance with such enhanced body weight gain, we found, using metabolic cages, that tau KI mice exhibited an increased food intake (Figure 2B) without modification of locomotor activity (Figure 2C) nor energy expenditure (Figure 2D). The respiratory exchange ratio (RER) remained unaltered suggesting that under HFD, tau KI mice do not exhibit major energy metabolism nor change of energy substrate oxidation at the tissue level (Figure 2E).

Interestingly, further investigations were indicative of glucose homeostasis disturbances in tau KI as compared to littermate WT mice under HFD. While 6-h-fasting or fed glycemia remained unaltered by HFD (Figures 2F,G), insulinemia (Figure 2H) as well as overnight fasting glycemia (Figure 2I) were found significantly enhanced in tau KI animals. Further, tau KI male mice fed under HFD also exhibited impaired glucose tolerance as assessed using the IPGTT (glucose tolerance) test (Figure 2J). Impaired glucose homeostasis was apparently not accompanied by peripheral insulin resistance since levels of liver pAkt, a downstream target of the insulin signaling pathway, remained similar between WT and tau KI animals (Supplementary Figure 4A). In line, levels of plasma adiponectin, an adipose tissue–secreted endogenous insulin sensitizer whose reduction is associated with insulin resistance, were not altered in tau KI mice (Supplementary Figure 4B).

**Figure 3:**
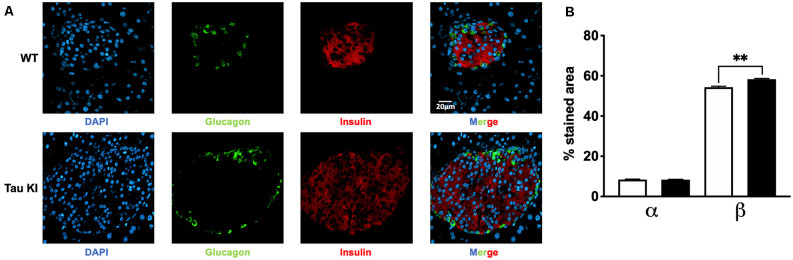
Analysis for both α and β cell fraction. **(A)** Representative double immunofluorescence staining for insulin (red) and glucagon (green) in pancreas sections from adult WT and tau KI mice. DAPI nuclear counterstaining was used (blue). Magnification for pancreatic sections = x40, scale bars = 20 μm. **(B)** Automated analysis of α and β cell fraction in pancreatic sections from tau KI and WT male mice under HFD (*N* = 1,521–1,883 islets from three mice/group; Kruskal Wallis test, *p* < 0.001; **p = 0.0096 using Dunn’s multiple comparisons test). Results are expressed as mean ± SEM. WT mice are indicated as white bars, tau KI mice as black bars.

Since tau KI animals have impaired glucose tolerance upon HFD, we next investigated whether insulin-producing β-cell mass could be altered. Indeed, increased β-cell mass may be indicative of an adaptive mechanism to impaired insulin secretion and therefore altered glucose homeostasis (Weir and Bonner-Weir, 2004). We thus evaluated the relative mass of α and β cells in pancreatic islets of WT and tau KI mice under HFD. Interestingly, enhanced insulinemia and glucose intolerance of tau KI mice under HFD were associated with an increased fraction of β-cell in the islets of tau KI mice as compared to WT littermates while glucagon-producing α-cells remained unaffected (Figure 3). The increase of β cell mass can be explained by the increase of both the number of pancreatic islets and the area of β-cells in KI mice. These data suggest that impaired glucose tolerance might relate to defective insulin secretion in response to glucose, more than a direct effect on beta cell mass.

Finally, to assess the possibility that hepatic glucose homeostasis could also be impaired in tau KI mice, animals were also challenged with pyruvate, a gluconeogenic precursor (Clementi et al., 2011). Pyruvate tolerance remained unaltered in tau KI mice as compared to their control littermates (Figure 2K) suggesting that liver glucose production was not associated with the glucose metabolic disturbances observed. Body temperature remained similar between genotypes (Figure 2L), possibly excluding gross thermogenesis alterations.

Importantly, we could uncover a sexual dimorphism in the glucose homeostasis impairments of tau KI mice since neither fasting glycemia, glucose tolerance nor body weight gain were affected in females KI mice under HFD as compared to their control littermates (Figures 2M–U). Altogether, the present data suggested that, when challenged with HFD, tau KI male mice exhibit significant glucose dyshomeostasis.

### Tau Is Expressed by Insulin-Producing Cells of Mouse and Human Islets

Changes in insulin levels, glucose tolerance, and changes in β-cell mass observed in KI male mice point towards a potential link between tau and function of pancreatic β cells.

**Figure 4:**
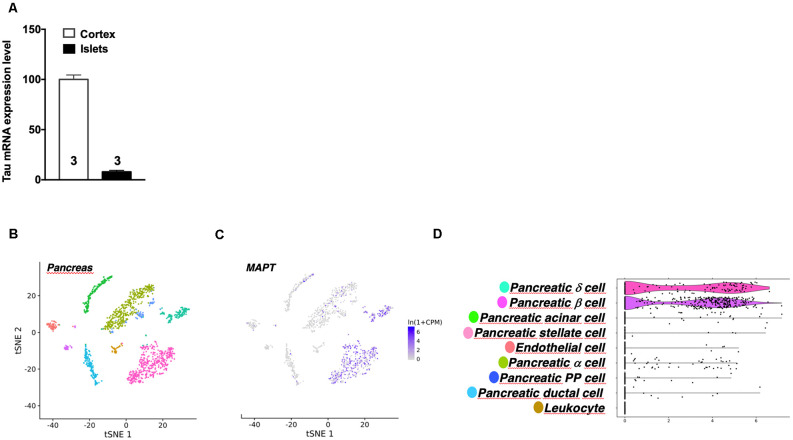
Tau mRNA expression in mouse pancreas. **(A)** Tau mRNA expression in pancreatic islets and cortex of adult wild type mice (5 months). Tau mRNA expression in endocrine pancreatic islets represents 10% of its expression in the hippocampus. Results are expressed as mean ± SEM. **(B)** t-Distributed Stochastic Neighbor Embedding (t-SNE) visualization of all mouse pancreatic cells collected by fluorescence-activated cell sorting (FACS). **(C)** Single-cell transcriptomic data of Mapt gene from adult mouse pancreatic tissue. **(D)** Differential expression of Mapt gene between different mouse pancreatic islets cells. For images **(B–D)** data were extracted from the Tabula Moris single-cell RNA-sequencing database.

It is noteworthy that while tau is particularly enriched in the brain, *Mapt* mRNA expression in pancreatic mouse islets represents 10% of its level in the cortex (Figure 4A). This is in line with public single-cell RNA sequencing data reporting tau mRNA enrichment in pancreatic β cells (but also δ cells) of pancreatic islets vs. α cells (Figures 4B–D; Segerstolpe et al., 2016). It is noteworthy that mouse islets expressed tau 4R isoforms (Supplementary Figure 5A) while human islets equally expressed both 3R and 4R tau isoforms (Supplementary Figure 5B), in agreement with brain expressions.

**Figure 5:**
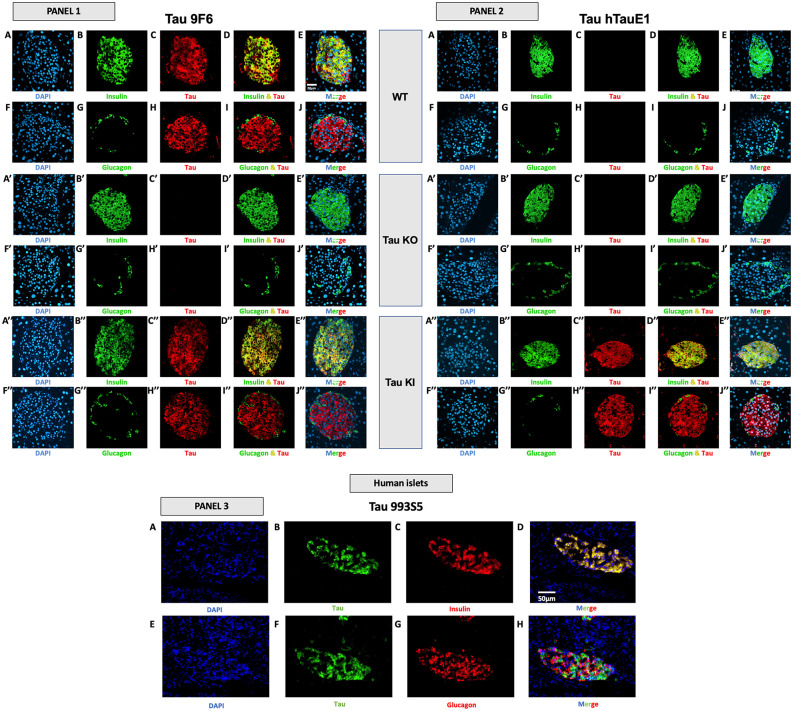
Tau expression in mouse and human pancreatic islets and colocalization with insulin and glucagon. **Panel 1 (9F6 antibody). Tau WT mice**. **(A–E)** Double immunofluorescence staining for **insulin** (green) and tau (red) in pancreatic islets from adult **WT** mice. Blue: DAPI nuclear counterstaining. **(F–J)** Double immunofluorescence staining for **glucagon** (green) and tau (red) in pancreatic islets from adult **WT** mice. Blue: DAPI nuclear counterstaining.** Tau KO mice (A’–E’)** Double immunofluorescence staining for **insulin** (green) and tau (red) in pancreatic islets from adult tau **KO** mice. Blue: DAPI nuclear counterstaining. **(F’–J’)** Double immunofluorescence staining for **glucagon** (green) and tau (red) in pancreatic islets from adult tau **KO** mice. Blue: DAPI nuclear counterstaining. **(K’–O’)**. **Tau KI mice (A”–E”)** Double immunofluorescence staining for **insulin** (green) and tau (red) in pancreatic islets from adult **KI** mice. Blue: DAPI nuclear counterstaining.** (F”–J”)** Double immunofluorescence staining for **glucagon** (green) and tau (red) in pancreatic islets from adult tau **KI** mice. Blue: DAPI nuclear counterstaining. These observations were reproduced in at least three independent experiments and samples. **Panel 2 tau expression using htauE1 antibody against human tau. Tau WT mice. (A–E)** Double immunofluorescence staining for **insulin** (green) and tau (red) in pancreatic islets from adult **WT** mice. Blue: DAPI nuclear counterstaining.** (F–J)** Double immunofluorescence staining for **glucagon** (green) and tau (red) in pancreatic islets from adult **WT** mice. Blue: DAPI nuclear counterstaining.** Tau KO mice (A’–E’)** Double immunofluorescence staining for **insulin** (green) and tau (red) in pancreatic islets from adult tau **KO** mice. Blue: DAPI nuclear counterstaining. **(F’–J’)** Double immunofluorescence staining for **glucagon** (green) and tau (red) in pancreatic islets from adult tau **KO** mice. Blue: DAPI nuclear counterstaining.** Tau KI mice (A”–E”)** Double immunofluorescence staining for **insulin** (green) and tau (red) in pancreatic islets from adult **KI** mice. Blue: DAPI nuclear counterstaining.** (F”–J”)** Double immunofluorescence staining for **glucagon** (green) and tau (red) in pancreatic islets from adult tau **KI** mice. Blue: DAPI nuclear counterstaining. Mice were 5 month-old. Scale: 20 μm. These observations were reproduced in at least three independent experiments and samples. **Panel 3 (tau 993S5 antibody). Human islets**. **(A–D)** Double immunofluorescence staining for tau (green) and **insulin** (red). Blue: DAPI nuclear counterstaining. **(E–H)** Double immunofluorescence staining for tau (green) and **glucagon** (red). Blue: DAPI nuclear counterstaining. Scale: 50 μm.

At the protein level, we investigated the expression of tau in the islets of WT and tau KI mice using various antibodies raised against the C-terminal (9F6) and the N-terminal (hTauE1) parts of tau (Figure 5, panels 1 and 2) as well as an antibody raised against the 162–175 amino-acids of tau (9H12; Supplementary Figure 6). We also evaluated the expression of tau in human islets (993S5 antibody; Figure 5, panels 3). In line with previous studies (Miklossy et al., 2010; Maj et al., 2010; Wijesekara et al., 2018b; Martinez-Valbuena et al., 2019), pancreatic islets from both WT and KI mice (9F6 and 9H12 antibodies; Figure 5, panel 1, C/H/C”/H” and Supplementary Figures 6C,H,C”,H”), as well as humans (Figure 5, panel 3, B/F), expressed tau protein. As expected, pancreatic islets from tau KI but not WT mice expressed human tau (hTauE1 antibody; Figure 5, panel 2, C/H vs. C”/H”). The specificity of the signal in mouse samples was attested by the lack of signal found in the pancreatic islets from tau KO mice (Figure 5, panels 1 and 2, C’/H’; Supplementary Figures 6C’,H’). The absence of signal was always observed when primary antibodies were omitted (data not shown and Supplementary Figures 6K–O,K’–O’,K”–O”).

Importantly, in both mouse and human pancreatic islets, tau protein was clearly expressed by β cells, expressing insulin (Figure 5, panel 1, A–E,A”–E”; panel 2, A”–E”; panel 3, A–D; Supplementary Figures 6A–E,A”–H”) but not by α cells, expressing glucagon (Figure 5, panel 1, F–J/F”–J”; panel 2, F”–J”; panel 3, E-H; Supplementary Figures 6F–J,F”–J”).

To corroborate these observations, we performed an overlapping quantification using the Pearson’s overlap coefficient as an index. The latter was determined using ImageJ Software on confocal Z-stacks. In WT mice (9F6 antibody), the Pearson coefficient for insulin/tau was found to be 0.74 ± 0.01 (*n* = 5) while values for glucagon/tau were extremely low 0.0005 ± 0.0006 (*n* = 5). In KI mice (hTauE1 antibody), the Pearson coefficient for insulin/tau was found to be 0.81 ± 0.02 (*n* = 5) while values for glucagon/tau were extremely low 0.02 ± 0.0004 (*n* = 5). In human islet (993S5 antibody), values were found to be 0.90 ± 0.06 (*n* = 3) for insulin/tau colocalization, while 0.18 ± 0.05 for glucagon/tau. Together, these data strongly support that in pancreatic islets, tau protein is largely enriched in β cells.

### Impaired Glucose-Stimulated Insulin Secretion in Isolated Islets From Tau KI and KO Mice

**Figure 6:**
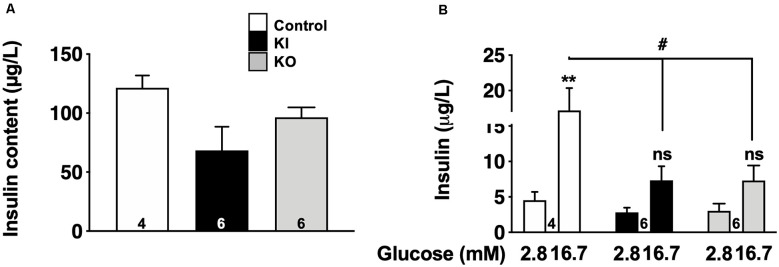
Glucose-stimulated insulin secretion (GSIS) on islets isolated from tau knock-in and knock-out mice. **(A)** Insulin content (μg/L) from isolated islets (*N* = 4–6 mice/group; NS using Kruskal Wallis test). **(B)** Insulin secretion from control, tau KI and tau KO islets stimulated with low (2.8 mM) and high (16.7 mM) glucose for 1 h (*N* = 4–6 mice/group; Two-Way ANOVA; *F*_(5,26)_ = 7.544 *p* < 0.001; Tukey’s *post hoc* test **p < 0.01, ^**#**^*p* < 0.05 vs. 2.8 mM). WT, tau KI, and tau KO are indicated as open bars, black bars and gray bars, respectively. Mice were 5-month-old. ns, non significant.

To fully appreciate the functional impact of tau loss-of-function on tau KI mice to β-cell function, we evaluated glucose-stimulated insulin secretion (GSIS) in low and high glucose conditions from pancreatic islets isolated from WT, tau KI mice, and tau KO male mice, taken as a control. Although the level of insulin expressed by islets was not significantly different in KO and KI mice vs. WT (Figure 6A), constitutive tau deletion or expression of the mutated form in KI significantly impaired insulin secretion upon 16.7 mM glucose stimulation (Figure 6B). Noteworthy, using the mouse pancreatic β-cell line Min6, we could also observe that tau knock-down by siRNA significantly impaired GSIS (Supplementary Figure 7). Taken together, these results support that loss of tau function (knock-down, KO, or KI) impairs insulin secretion in response to glucose without affecting insulin content, suggestive of a direct effect of tau loss-of-function on insulin secretion rather than insulin biogenesis.

## Discussion

The origin of glucose homeostasis alterations in AD but also FTLD patients remains unclear (Bucht et al., 1983; Fujisawa et al., 1991; Craft et al., 1992; Janson et al., 2004; Matsuzaki et al., 2010; Ahmed et al., 2014; Calsolaro and Edison, 2016; Tortelli et al., 2017). Previous works, from our laboratory and others, using germline tau KO mice, supported that constitutive loss of tau function can lead to glucose homeostasis impairments (Marciniak et al., 2017; Wijesekara et al., 2018b, 2021). These data however are not sufficient to determine whether these changes solely relate to tau deletion, which is pathophysiologically irrelevant, or to the loss of some tau functions. To address more specifically this question, the principal microtubule-binding property of tau can be reduced by the insertion of mutations in the microtubules domains such as those described in FTLD with tau mutations. Therefore, to further address the link between tau and glucose homeostasis, we, therefore, used a novel knock-in tau mouse model expressing a mutated human tau protein, under the endogenous *Mapt* mouse gene promoter, allowing expression of a mutated human tau protein at a physiological level and thereby avoiding the biases of mouse models based on tau-overexpression (Leboucher et al., 2019) or constitutive deletion. This model was chosen to determine to which extent a loss of tau microtubule-binding activity due to P301L mutation (Delobel et al., 2002) was prone to recapitulate metabolic impairments observed in tau KO animals. These investigations were performed at an age when the model does not exhibit any tau aggregation allowing us to evaluate the impact of the expression of a dysfunctional tau protein.

Our data demonstrate that expressing a mutated form of tau favors the development, in males, of glucose homeostasis impairments under metabolic stress (HFD), as exemplified by the significant increase in insulinemia as well as impaired glucose tolerance. The metabolic phenotype observed in tau KI mice under HFD mirrored what we and others previously observed in constitutive tau KO animals (Marciniak et al., 2017; Wijesekara et al., 2018b, 2021), likely suggesting that disturbances observed in tau KI and tau KO mice are likely ascribed to an impaired tau function such as the loss of microtubule-binding activity. This view is in agreement with the reversion of tau KO phenotype observed following re-expression of human tau as recently reported (Wijesekara et al., 2021). Nonetheless, glucose homeostasis defects have been observed in another tau KI model, where mutated tau sequence is inserted in the permissive HPRT site and expressed at the physiological level in the presence of murine tau, suggesting that metabolic dysregulations are not solely ascribed to tau loss-of-function and that mechanistic insights on the precise role of tau in the control of glucose homeostasis require additional molecular studies.

Our data support that glucose metabolism impairments seen in tau KI mice clearly involve pancreatic islets dysfunction rather than insulin resistance. To evaluate insulin resistance, we analyzed phosphorylated serine/threonine kinase protein kinase B (pAkt) and circulating adiponectin, two key molecular parameters that are impaired during insulin resistance. Indeed, insulin signaling in the liver or adipose tissue induces the phosphorylation of Akt and its subsequent activation. In several models of insulin resistance, it has been shown that reduced glucose uptake is due to defects in insulin signaling (Ng et al., 2010; Huang et al., 2018) and is associated with impaired Akt phosphorylation, leading to the development of insulin resistance in obesity and type 2 diabetes (Choi and Kim, 2010). Adiponectin is an adipose tissue-secreted endogenous insulin sensitizer, which plays a key role as a modulator of peroxisome proliferator-activated receptor γ action. Low levels of adiponectin, as observed in AdipoQ knockout mice or in patients affected by type 2 diabetes, have been associated with insulin resistance in diabetes (Ziemke et al., 2010). In our study, both pAkt and serum adiponectin concentrations were not significantly different between KI and WT mice fed with HFD, suggesting that insulin sensitivity was not impaired in our model. However, we provide clear evidence of the large enrichment of tau in insulin-producing β cells in WT, tau KI mice as well as the human pancreas. Second, we observed, in tau KI mice, a significant increase of β-cell mass (Figure 3) similar to what was previously reported during hyperglycemia and/or insulin resistance (Weir and Bonner-Weir, 2004). Third, this adaptation might relate to the impaired insulin secretion we observed in *ex vivo* experiments on isolated pancreatic islets; itself linked to a loss-of-function of mutated tau, these observations being replicated in isolated islets from tau KO mice as well as from tau knock-down in Min6 pancreatic cell line. Although it was already known that pancreas and islets of Langerhans expressed tau mRNA (Vanier et al., 1998; Maj et al., 2010), our data are the first reporting that isoforms expressed are a mix of 3R/4R or 4R only, in human and mouse islets respectively, in agreement with the brain profile. Previous studies suggested the presence of tau protein into human pancreatic β cells (Miklossy et al., 2010; Martinez-Valbuena et al., 2019). Our study is also the first to report the colocalization of tau with insulin but not glucagon in human islets. Other studies suggested that, in rat or mouse islets, tau colocalizes with insulin (Maj et al., 2010; Wijesekara et al., 2018b). Extending these primary findings, our data unambiguously show, using several tau antibodies and proper controls (tau KO tissue combined with confocal microscopy) that, in the mouse, tau is selectively expressed by insulin but not glucagon-positive cells. Such demonstration was also important given a recent article (Zhou et al., 2020) suggesting that tau might not be expressed by pancreatic islets.

*In vitro* GSIS from isolated pancreatic islets from tau KO mice, consistent with previous observations (Wijesekara et al., 2018b), or tau KI mice, showed an impaired insulin secretion upon high glucose conditions. Altered GSIS was however not associated with a defective insulin production and/or decreased β-cell mass. Therefore, the machinery that controls insulin secretion in response to glucose is impaired in tau KI mice, probably contributing to hyperglycemia observed in these mice. In the context of glucose dyshomeostasis, such as during type 2 diabetes development, it is not fully inconsistent to observe both defective insulin secretion and increased fasting hyperglycemia and insulinemia (DeFronzo et al., 2015). Increased fasting plasma insulin levels, observed in tau KI mice, can be caused by a compensatory mechanism induced by hyperglycemia that leads to an increase of β-cell mass (Weir and Bonner-Weir, 2004). In addition, reduced insulin clearance is observed during HFD feeding and can contribute to maintaining elevated fasting insulinemia (Strömblad and Björntorp, 1986). Interestingly, studies in humans (Bonora et al., 1983) and animals (Kim et al., 2007) have shown that reduced insulin clearance can cooperate with elevated insulin secretion to regulate glucose homeostasis.

How tau regulates the ability to secrete insulin in response to high glucose remains unclear. Notably, microtubules play a major role in the intracellular trafficking of vesicles in endocrine cells like pancreatic β cells (Fourriere et al., 2020; Müller et al., 2021). A recent study demonstrated that high levels of glucose induce rapid microtubule disassembly mediated by tau hyperphosphorylation *via* glucose-responsive kinases, leading to tau dissociation from microtubules and favoring insulin secretion (Ho et al., 2020). Furthermore, in line with our finding, it was reported recently that tau knockdown in mouse pancreatic β cells facilitated microtubule turnover, causing an increase of basal insulin secretion, depleting insulin vesicles from the cytoplasm, which subsequently impaired GSIS (Ho et al., 2020). Hence, in β cells, tau plays an important role in glucose-mediated insulin secretion. Considering that tau is more than a microtubule-associated protein (Sotiropoulos et al., 2017), and plays a role in chromatin organization and RNA metabolism (Galas et al., 2019), it is also possible that the impaired tau function also alters ß cell function by other mechanisms.

An important observation of the present study is the sexual dimorphism in the ability of tau to regulate glucose homeostasis, with male tau KI mice being significantly more impacted than female littermates. Until then, previous works investigating the metabolic outcomes of tau deletion were only performed in males (Marciniak et al., 2017; Wijesekara et al., 2018b, 2021). Sex is known to impact the response to metabolic stress and β-cell engagement. Like in humans, where women are less likely than men to develop type 2 diabetes (Kautzky-Willer et al., 2016), female mice are more resistant to HFD than males (Oliveira et al., 2015) and manifest improved glucose tolerance, with greater insulin sensitivity in liver, muscles and adipose tissue (Goren et al., 2004). Conversely, male rodents exhibit a greater propensity for β cell failure (Gannon et al., 2018). Increased estrogen receptor signaling, differences in islet DNA methylation status, expression differences of antioxidant genes and of islet-enriched genes transcription factors have all been suggested as causes for these differences allowing females to tolerate HFD better than males (Liu and Mauvais-Jarvis, 2010; Osipovich et al., 2020 and references herein). In accordance, another important point that has not been addressed in the present study is the potential sexual-dimorphism of insulin secretion by isolated islets in response to glucose. Therefore the sex-related differences we uncovered in tau KI mice could be likely due to the action of sex hormones but also estrus cycle issues that will need to be further investigated. These data also highlight that tau is dispensable into the mechanisms underlying the protective influence of female hormones in mice.

Considering the presence of hyperphosphorylated and misconformed tau in the pancreatic islets of patients with type 2 diabetes and patients with AD (Miklossy et al., 2010; Martinez-Valbuena et al., 2019), it is likely that glucose homeostasis impairments seen in latter are, at least in part, related to tau loss of microtubule-binding activity. Nonetheless, it is probable that glucose metabolism impairments of AD patients likely arise from a synergistic impact of both pancreatic tau pathology and amyloidosis. Indeed, Aβ has been shown to deposit in the pancreas of both humans and APP mouse models (Miklossy et al., 2010; Vandal et al., 2014; Wijesekara et al., 2017). This is in agreement with the glucose homeostasis impairments seen in the latter (Takeda et al., 2010; Mody et al., 2011; Vandal et al., 2015; for review see Wijesekara et al., 2018a). Regarding AD patients but also individuals with FTLD, we cannot rule out that besides β cells, other peripheral organs or brain structures are involved in their glucose metabolism impairments. Indeed, tau is physiologically expressed by skeletal muscle or kidney (Gu et al., 1996; Caillet-Boudin et al., 2015; https://www.proteinatlas.org/ENSG00000186868-MAPT/tissue), both involved in glucose homeostasis (Gerich, 2010; Triplitt, 2012), even if, to the best of our knowledge, tau hyperphosphorylation/misconformation has not been described at these locations. However, tau misconformation, and therefore tau loss-of-function, is well observed in brain regions such as the hippocampus and hypothalamus, known to control glucose homeostasis (Schultz et al., 1999; Ishii and Iadecola, 2015; Soto et al., 2019). The relative contribution of the pancreas and the brain area in controlling peripheral glucose homeostasis warrants further investigations using tissue-specific conditional expression approaches allowing cell-specific loss of tau function.

Finally, regardless of whether pancreatic β cells or brain area are primarily involved in glucose metabolism impairments seen in AD and FTLD patients, considering that diabetes and impaired glucose tolerance are important risk factors for both (Reitz et al., 2011; Golimstok et al., 2014; Livingston et al., 2017) and that both exacerbate learning and memory defects and underlying pathology in different models reproducing the amyloid and tau lesions (Takeda et al., 2010; Leboucher et al., 2013; for review see Wijesekara et al., 2018a), glucose metabolism deficits promoted by both tau and amyloid lesions would then be part of a detrimental circle that would ultimately favor cognitive decline. Moreover, such a mutual relationship between glucose homeostasis disturbance and AD, with probably common pathophysiological mechanisms, requires a change in public health policies by focusing more on primary prevention of common risk factors for diabetes and AD. General public awareness is needed about the risk of developing these two diseases, and the importance of correcting modifiable risk factors, such as healthier eating, weight loss, and increased physical activity. Furthermore, investigating and pharmacologically managing glucose homeostasis deficits at an early pathological stage of AD or FTLD patients would be then of clinical interest in cross-consultations between neurology and endocrinology departments.

In summary, the present study highlights that knock-in expression of a mutated tau protein favors the development of glucose metabolism impairments and pancreatic β-cell dysfunction upon metabolic stress, supporting not only a role of tau pathology in the development of metabolic disturbances in AD and FTLD patients but providing new insights on the physiological role of tau in the control of peripheral metabolism.

## Data Availability Statement

The raw data supporting the conclusions of this article will be made available by the authors, without undue reservation.

## Ethics Statement

The studies involving human participants were reviewed and approved by French Agency for Biomedical Research (Agence de la Biomedecine, Saint-Denis la Plaine, France, protocol no. PFS16-002) and the Lille Neurobiobank (DC-2008-642). The patients/participants provided their written informed consent to participate in this study. The animal study was reviewed and approved by CEEA75.

## Author Contributions

Conceptualization: HB, AM-T, SL, KB, NS, J-SA, LB, DV, DB, and VB-S. Experiments: HB, SK, ECa, SE, CB, EF, LR, ECo, MB, FO, TB, KC, BT, TG, EN, VG-M, ABon, and ABog. Data analysis: HB, SK, EC, SE, CB, J-SA, DV, DB, and NS. Funding acquisition: J-SA, LB, and DB. Supervision: J-SA, LB, DV, DB, and VB-S. Writing—original draft: HB, SK, J-SA, DV, DB, LB, and VB-S. Writing—review and editing: all. All authors contributed to the article and approved the submitted version.

## Conflict of Interest

The authors declare that the research was conducted in the absence of any commercial or financial relationships that could be construed as a potential conflict of interest.

## Publisher’s Note

All claims expressed in this article are solely those of the authors and do not necessarily represent those of their affiliated organizations, or those of the publisher, the editors and the reviewers. Any product that may be evaluated in this article, or claim that may be made by its manufacturer, is not guaranteed or endorsed by the publisher.

## Footnotes

^1^ https://www.biochain.com/product/paraffin-tissue-section-human-adult-normal-pancreas/
