# Supplementary material for: Impaired Glucose Homeostasis in a Tau Knock-In Mouse Model
Source: Front Mol Neurosci. 2022 Feb 16;15:841892. doi: 10.3389/fnmol.2022.841892 (PMC8889017; doi:10.3389/fnmol.2022.841892)
Supplement: Supplementary file 1 [file Table_1.pdf]

Primer used	Annealing T°C Cycles	Primer sequence	PCR products	Products length (bp)
**Mouse tau exon 10 alternative splicing**				
TMF1<br>TMR1	59°C<br>35 cycles	CTG AAG CAC CAG CCA GGA GG<br>CGA TGC TGC CCG TGG AGG AGA		
TMF1<br>TRR2	59°C<br>35 cycles	CTG AAG CAC CAG CCA GGA GG<br>GTC TGT CTT GGC TTT GGC ATT CTC	4R (10+)<br>3R (10-)	600<br>500
**Human tau exon 10 alternative splicing**				
Forward<br>Reverse	60°C<br>30 cycles	CAT GCC AGA CCT GAA TGT CAA G<br>TCA CAA ACC CTG CTT GGC CA	4R (10+)<br>3R (10-)	244<br>151
