# Supplementary material for: Impaired Glucose Homeostasis in a Tau Knock-In Mouse Model
Source: Front Mol Neurosci. 2022 Feb 16;15:841892. doi: 10.3389/fnmol.2022.841892 (PMC8889017; doi:10.3389/fnmol.2022.841892)
Supplement: Supplementary file 2 [file Image_1.pdf]

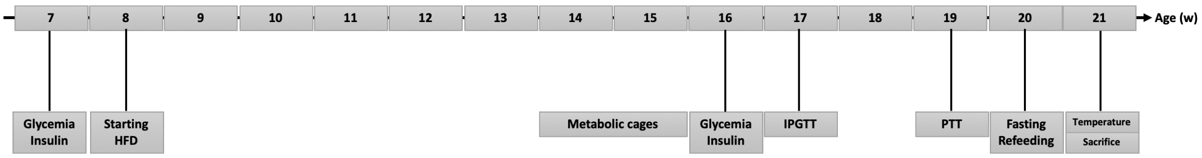

Supplementary Figure 1. Time-line of metabolism investigations in tau KI and littermate WT animals under high-fat diet. Baseline glycemia and plasma insulin measurement were performed after 6 hours of fasting and collected at the age of 7 weeks. Feeding with high-fat diet was started at 8 weeks old. Following metabolic cage measurements (6-7 weeks after HFD onset), metabolic exploration was performed from 8 to 12 weeks following HFD onset. Glycemia and plasma insulin measurements were determined 8 weeks after HFD onset (16 weeks of age). Intraperitoneal glucose tolerance tests (IPGTT) was performed at the 9th week of HFD (17 weeks old). Pyruvate tolerance test (PTT) after an overnight fasting was performed at the 11th week of HFD (19 weeks old). Glycemia measurement after overnight fasting and during the first 4 hours of refeeding was performed at the 12th week of HFD (20 weeks old). At the 13th week of HFD rectal temperature was measured and animals sacrificed.

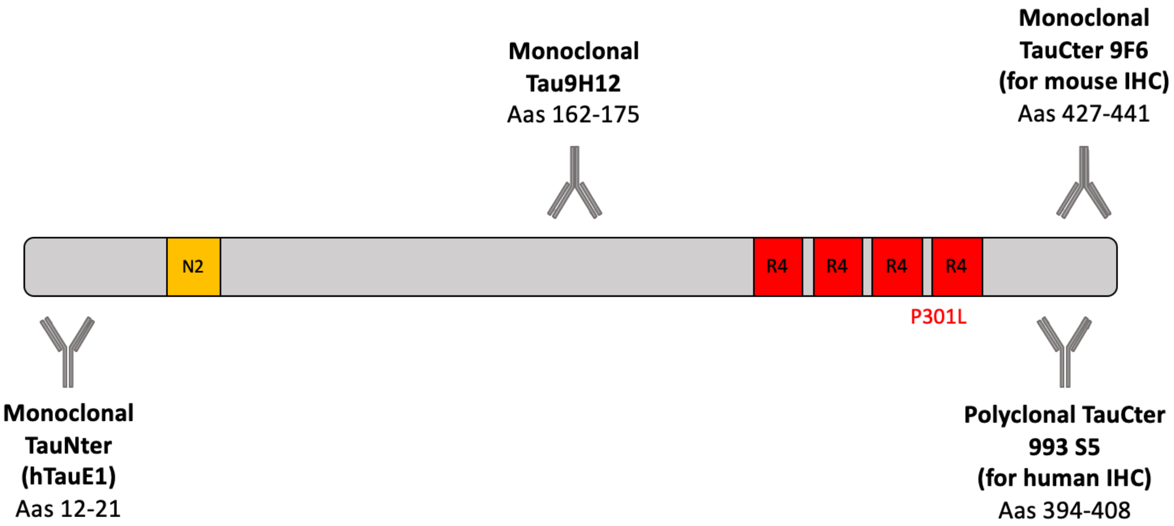

Supplementary Figure 2. Home-made antibodies used for tau immunohistochemistry and Western blots with respective epitopes. Tau 9H12, tauCter 9F6/993 S5 and tauNter htauE1 targeting epitopes are represented. Epitopes are shown with their associated sites corresponding to the longest isoform of tau protein.

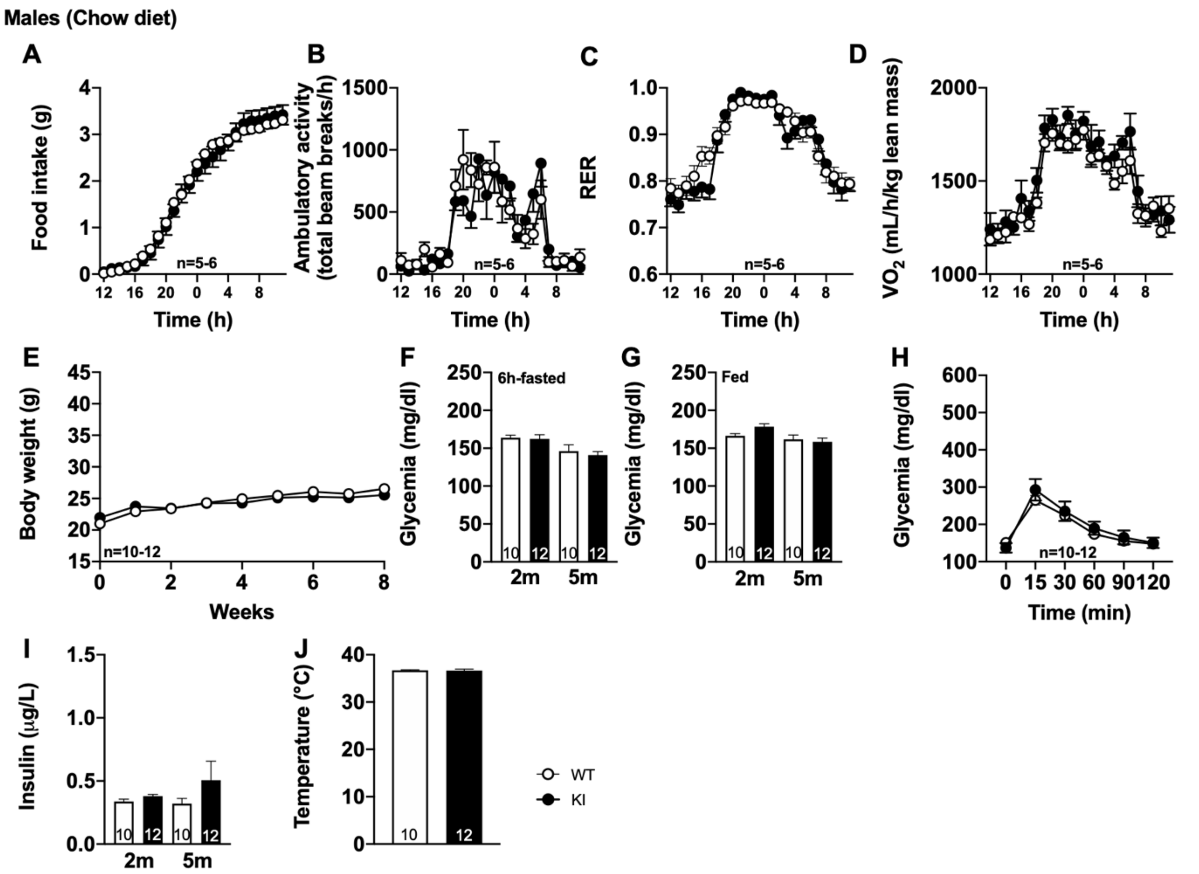

Supplementary Figure 3. Metabolic phenotyping of tau KI male mice under chow diet. (**A-D**) Metabolic cage evaluation of tau KI male mice under chow diet. (**A**) 24h-cumulative food intake (g) (NS). (**B**) 24h spontaneous locomotor activity (total beam breaks/h) (NS). (**C**) 24h-respiratory exchange ratio (RER=VCO2/VO2) (NS). (**D**) 24h-O_2 consumption (NS). (**E**) Body weight gain of WT and tau KI mice from 2 to 5 months of age (NS). (**F**) Glycemia after 6 hours of fasting at 2 to 5 months of age (NS). (**G**) Glycemia in fed condition (9.a.m) at 2 to 5 months of age (NS). (**H**) Intraperitoneal glucose tolerance test (IPGTT) at 5 months of age (NS). (**I**) Insulinemia after 6 hours of fasting at 2 to 5 months of age (NS). (**J**) Rectal temperature at 5 months of age (NS). Results are expressed as mean ± SEM. WT mice are indicated as white circles/bars, tau KI mice as black circles/bars.

A

B

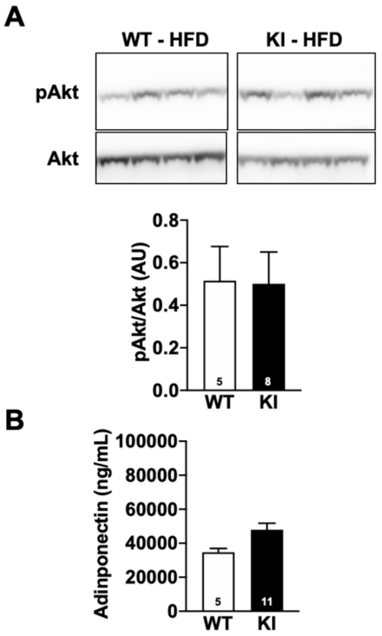

Supplementary Figure 4. (**A**) pAkt and Akt protein expression in the liver of WT and KI mice under HFD (NS Student's t-test). Western blots are representative. (**B**) Adiponectin dosage in the plasma of WT and KI mice under. WT and KI are indicated as open bars and black respectively. Mice were 5 months old.

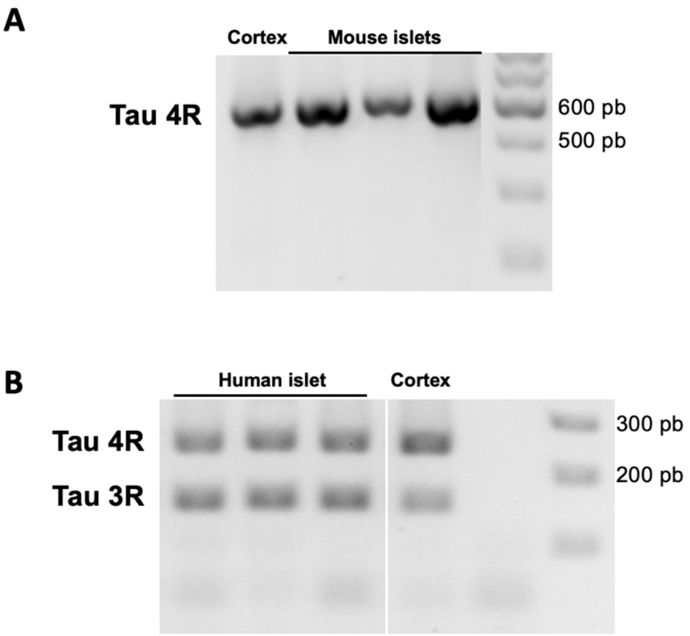

Supplementary Figure 5. PCR analysis of mouse and human tau exon 10 splicing in mouse and human pancreatic islets. (**A**) Mouse analysis in pancreatic islets (n=3) vs. cerebral cortex (n=1). Mice were 5 months old. (**B**) Human analysis in pancreatic islets (n=3) vs. cerebral cortex (n=1).

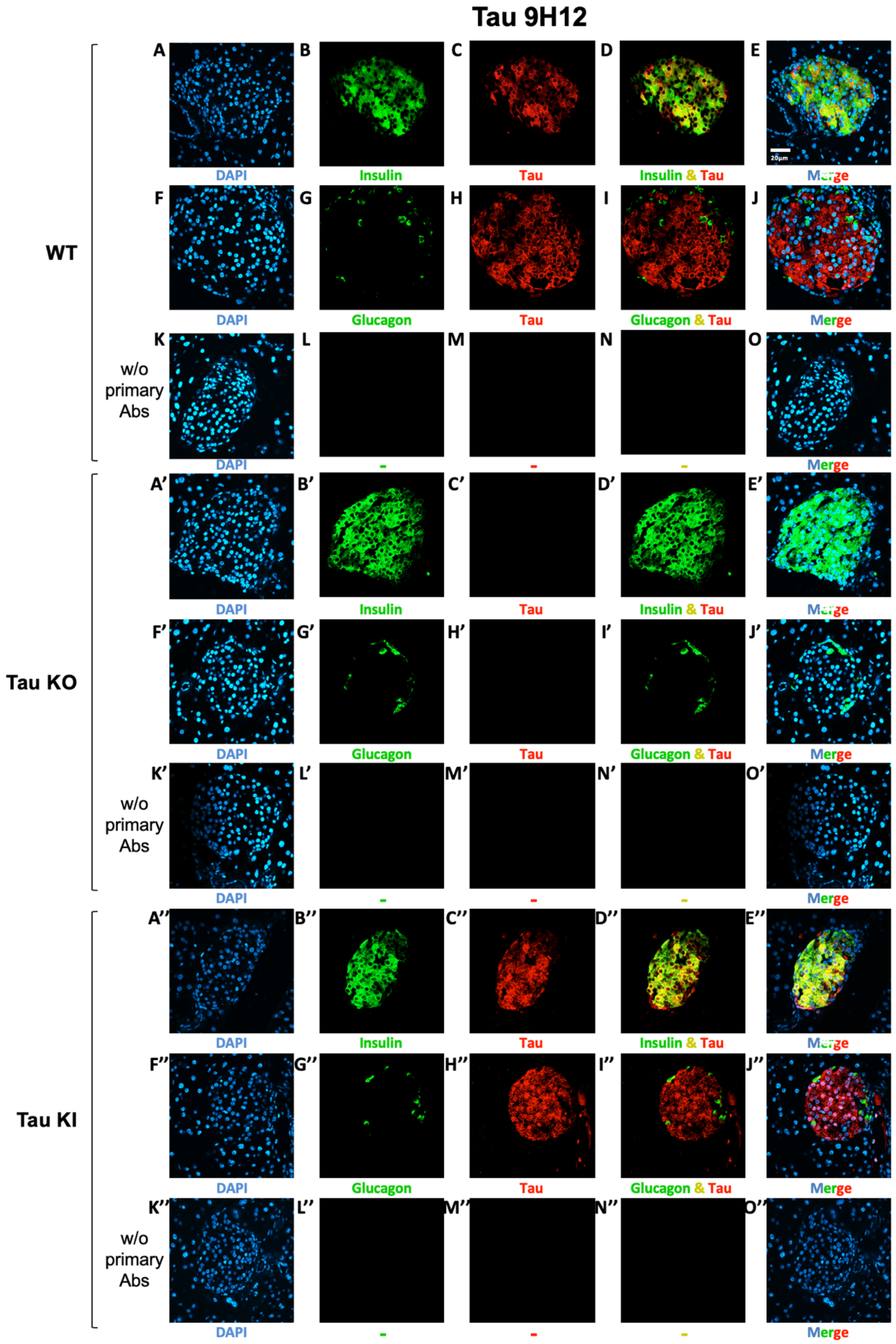
Tau 9H12
WT
Tau KO
Tau KI
A
B
C
D
E
20µm
DAPI
Insulin
Tau
Insulin & Tau
Merge
F
G
H
I
J
DAPI
Glucagon
Tau
Glucagon & Tau
Merge
K
L
M
N
O
w/o primary Abs
DAPI
Merge
A'
B'
C'
D'
E'
DAPI
Insulin
Tau
Insulin & Tau
Merge
F'
G'
H'
I'
J'
DAPI
Glucagon
Tau
Glucagon & Tau
Merge
K'
L'
M'
N'
O'
w/o primary Abs
DAPI
Merge
A''
B''
C''
D''
E''
DAPI
Insulin
Tau
Insulin & Tau
Merge
F''
G''
H''
I''
J''
DAPI
Glucagon
Tau
Glucagon & Tau
Merge
K''
L''
M''
N''
O''
w/o primary Abs
DAPI
Merge

Supplementary Figure 6. Tau expression (tau 9H12 antibody) in mouse islets from WT, tau KO and tau KI mice and colocalization with insulin and glucagon.

Tau WT mice. (A-E) Double immunofluorescence staining for **insulin** (green) and tau (red) in pancreatic islets from adult **WT** mice. Blue: DAPI nuclear counterstaining. **(F-J)** Double immunofluorescence staining for **glucagon** (green) and tau (red) in pancreatic islets from adult **WT** mice. Blue: DAPI nuclear counterstaining. **(K-O)** Absence of staining without primary antibodies. Only secondary antibodies and DAPI nuclear counterstaining (blue) were used.

Tau KO mice (A'-E') Double immunofluorescence staining for **insulin** (green) and tau (red) in pancreatic islets from adult tau **KO** mice. Blue: DAPI nuclear counterstaining. **(F'-J')** Double immunofluorescence staining for **glucagon** (green) and tau (red) in pancreatic islet from adult tau **KO** mice. Blue: DAPI nuclear counterstaining. **(K'-O')** Absence of staining without primary antibodies. Only secondary antibodies and DAPI nuclear counterstaining (blue) were used.

Tau KI mice (A''-E'') Double immunofluorescence staining for **insulin** (green) and tau (red) in pancreatic islets from adult **KI** mice. Blue: DAPI nuclear counterstaining. **(F''-J'')** Double immunofluorescence staining for **glucagon** (green) and tau (red) in pancreatic islet from adult tau **KI** mice. Blue: DAPI nuclear counterstaining. **(K''-O'')** Absence of staining without primary antibodies. Only secondary antibodies and DAPI nuclear counterstaining (blue) were used. Mice were 5 month-old. Scale: 20μm. These observations were reproduced in at least 3 independent experiments and samples.

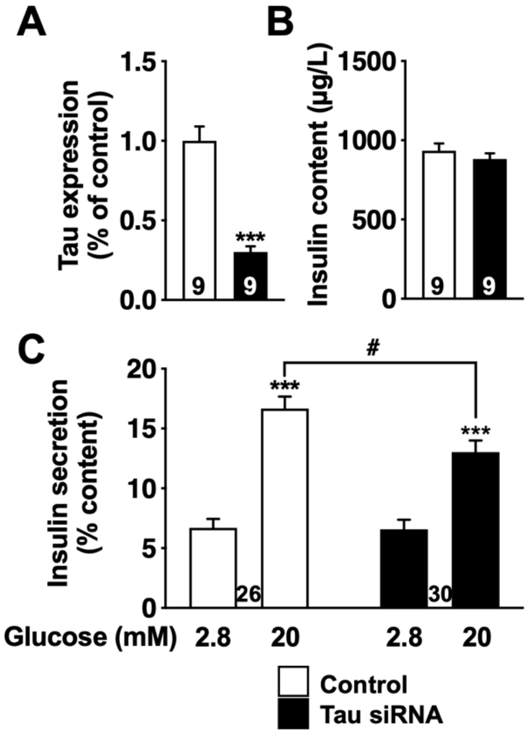

Supplementary Figure 7. Effect of tau knock-down on insulin secretion at glucose stimulated insulin secretion (GSIS) in β pancreatic mouse line Min6. (**A**) Tau mRNA expression level in control and tau siRNA conditions (***p< .0001, Student's t-test). (**B**) Min6 cells insulin content at GSIS (NS, Student's t-test). (**C**) Effect of tau knock-down on insulin release by Min6 cells in low and high glucose conditions at GSIS (Two-Way ANOVA, $F(1,108)=4.39$, $p<0.05$ test; Tukey's post-hoc test ***$p<0.0001$ vs 2.8mM; # $p<0.05$).
